# Alkaline phosphatase interference in immuno-enzymatic assays

**DOI:** 10.5937/jomb0-33981

**Published:** 2022-07-29

**Authors:** Osman Oğuz, Huriye Serin, Fatma Sinem Hocaoglu

**Affiliations:** 1 Istanbul Education and Research Hospital, Department of Medical Biochemistry, Istanbul, Turkey; 2 Düzen Lab, Department of Clinical Biochemistry, Istanbul,Turkey

**Keywords:** Alkaline phosphatase, ALP, bias, immunoenzymatic, total error, alkalna fosfataza, ALP, pristrasnost, imuno-enzimski, ukupna greška

## Abstract

**Background:**

Alkaline phosphatase (ALP) enzymes are widely used as signal amplifiers in immunoenzymatic methods. Conditions that cause ALP elevations, such as bone or liver diseases, can cause interference in immunoenzymatic methods. We aimed to examine ALP's effect on immunoenzymatic assay by adding isolated pure ALP to the prepared serum pool.

**Methods:**

We prepared a serum pool and divided it into 4 groups. By adding isolated pure ALP at different concentrations to each group, we obtained sample groups containing ALP enzyme at concentrations of 85 U/L, 340 U/L, 870 U/L, and 1570 U/L. 20-repetition of bhCG, ferritin, FT4, TSH, troponin I, and Vit B12 tests were performed in each group. The coefficient of variation, bias, and total error was calculated. All groups were compared by using the Friedman test for paired samples.

**Results:**

After ALP addition, the calculated total error values of FT4, bhCG and troponin I tests were above the acceptable error limits. There were statistically significant differences in bhCG, FT4, troponin I, and Vit B12 tests compared to the baseline ALP level (P<0.0125).

**Conclusions:**

Isolated ALP elevations can be a source of interference for immunoenzymatic methods.

## Abstract (sr)

**Uvod:**

Enzimi alkalne fosfataze (ALP) se široko koriste kao pojačivači signala u imunoenzimskim metodama. Stanja koja izazivaju povišenje ALP, kao što su bolesti kostiju ili jetre, mogu izazvati smetnje u imunoenzimskim metodama. Naš cilj je bio da ispitamo efekat ALP-a na imuno enzimski test dodavanjem izolovanog čistog ALP-a u pripremljenu grupu uzoraka seruma.

**Metode:**

Pripremili smo veći broj uzoraka seruma i podelili ih u 4 grupe. Dodavanjem izolovanog čistog ALP u različitim koncentracijama svakoj grupi, dobili smo grupe uzoraka koje sadrže ALP enzim u koncentracijama od 85 U/L, 340 U/L, 870 U/L, i 1570 U/L. Urađeno je 20 ponavljanja u svakoj grupi testova za bhCG, feritin, FT4, TSH, troponin I, i Vit B12. Izračunati su koeficijent varijacije, pristrasnost i ukupna greška. Sve grupe su upoređene korišćenjem Fridmanovog testa za uparene uzorke.

**Rezultati:**

Nakon dodavanja ALP, izračunate ukupne vrednosti greške testova FT4, bhCG i troponina I su bile iznad prihvatljivih granica greške. Postojale su statistički značajne razlike u testovima za bhCG, FT4, troponin I i Vit B12 u poređenju sa osnovnim nivoom ALP (P<0,0125).

**Zaključak:**

Izolovana povišenja ALP mogu biti razlog smetnji za sprovođenje imunoenzimskih metoda.

## Introduction

Alkaline phosphatases (ALP; orthophosphate mono-ester phosphohydrolase (alkaline optimum) EC 3.1.3.1) are homodimeric and glycoprotein enzymes in the hydrolase group with a molecular weight of 86 kilodaltons. These groups of enzymes are commonly found in nature in both eukaryotes and most prokaryotes. Each monomer is encoded by multiple genes to contain five cysteine residues, two zinc atoms and one magnesium atom, which are vitally important for its catalytic function.

ALP enables the detachment of phosphate groups from a variety of molecules, including nucleotides, proteins and alkaloids, in alkaline pH environments [1].

ALP mainly functions as bound by hydrophobic glucosyl-phosphatidylinositol to the cell membrane [2]. It is mostly found in the canalicular membrane of hepatocytes and bile duct epithelium lumen, bone osteoblasts, brush border membrane of the intestinal mucosa, placenta, proximal kidney tubules, and breast tissue during lactation. A healthy human serum contains four different ALP isoenzymes under normal conditions. These are Intestinal ALP, Placental ALP, Germ cell ALP and tissue nonspecific alkaline phosphatase. The difference between the isoenzymes stem from the sialic acid found in them at varying rates, and also, the protein amount of the placental isoenzyme is different [2] [3].

Serum ALP measurement plays a vital role in the diagnosis of hepatobiliary and bone diseases associated with an increased osteoblastic activity. Intrahepatic and extrahepatic cholestasis, spaceoccupying lesions in the liver, metastasis and infiltrative liver diseases cause an increase in serum ALP levels [4]. Apart from the liver diseases, serum ALP levels elevate as a result of Paget's disease (associated with increased osteoblastic activity), osteomalacia and rickets associated with vitamin D deficiency, primary and secondary hyperparathyroidism, and during the healing process of bone fractures. Likewise, placenta-induced ALP enzyme elevation is seen in the third trimester of pregnancy and placental or germ cell malign diseases [4].

ALP is used to provide signal amplification by conjugating it with antibodies in immunoenzymatic methods [4] [5] [6]. ALP is frequently used in immunochemical methods along with Horseradish Peroxidase due to its substrate diversity, cheapness and accessible possibility. Once conjugated with antibodies, antigens and streptavidin, these enzymes increase the test sensitivity owing to their low background effect, linear reaction rate, and extended incubation time. Using ALP as a signal amplifier provides an enhanced and longer-lasting lumination obtained at the end of the reaction. Thus, the target analyte can be assayed in lesser concentrations and much broader linearity. It was reported that while the endogenous ALP had an interfering effect on immunochemical methods in the previous automated systems, this effect is prevented through the increased washing processes in the renewed automated systems [4] [7].

In our laboratory, we use UniCelDxl 800 and Access II (Beckman Coulter, Brea, CA) auto analyzers that measure by immunoenzymatic method and use ALP conjugates as signal amplifiers. Our purpose is to evaluate the interference caused by the ALP elevation on these systems through beta human chorionic gonadotropin (βhCG), ferritin, free thyroxine (FT4), thyroid-stimulating hormone (TSH), troponin I and Cobalamin (Vit B12) tests.

## Materials and methods

We created a serum pool with 20 patients' sera who have previously consulted our laboratory in January 2020 and whose ALP, βhCG, ferritin, FT4, TSH, troponin I, and Vit B12 tests were found to be within the reference range.

The prepared serum pool was divided into four groups. Commercially prepared isolated ALP (Toyoba Enzymes, Osaka, Japan), with an activity of 30,000,000 U/L, was added at different doses to the groups. Commercial ALP enzyme is in transparent liquid form, has grade 2 activity (30,000 U/mL or more) and contains Adenosine deaminase and Phosphodiesterase. First of all, due to the enormous enzyme activity, isolated ALP was diluted to a working stock by adding 2 μL of commercial ALP to 998 μL of distilled water. We prepared a stock solution immediately before the assay. We added 5 μL, 10 μL and 20 μL of this stock solution, respectively, to the serum pools of 8 mL each. We obtained four groups, one being our reference group without ALP addition and the others containing ALP at concentrations of 340 U/L, 870 U/L and 1590 U/L after adding isolated ALP.

### Biochemical analysis

The ALP was measured spectrophotometrically with a Beckman Coulter AU 5800 (Beckman Coulter, Brea, CA) auto-analyzer. Ferritin, FT4, TSH and Vit B12 tests were measured immunoenzymatically with an UniCelDxl 800 (Beckman Coulter, Brea, CA) autoanalyzer and βhCG and troponin I test with an Access II (Beckman Coulter, Brea, CA) auto-analyzer. All assays are two-site sandwich immunoassay using enzyme-conjugated antibodies with direct chemiluminometric technology. In all groups, each test was performed with 20 repetitions. All measurements were completed on the same day and within-run.

### Statistical analysis

In the evaluation of the effect of the ALP enzyme on the immunoenzymatic tests, Total error (TE), Bias and coefficient of variations (CV) were calculated by 20 repetitions of each group.

TE was calculated by the following formula:


(1) \text{TE} =  │\text{Bias} \ \% │+ 2\text{CV}

TE = total error

CV = coefficient of variation.

Bias was calculated as follows [8]:


(2) \text{Bias} \ \ \%  =(\frac{C_2-C_1}{C_1}) \times 100

C_1_ = mean value of reference group and C_2_ =mean value of groups added with ALP

We applied the Friedman test to show the difference between groups using Med Calc (MedCalc Software Mariakerke, Belgium) software. We used Bonferroni correction to determine the adjusted significance level as p<0.0125.

## Results

**Figure 1:**
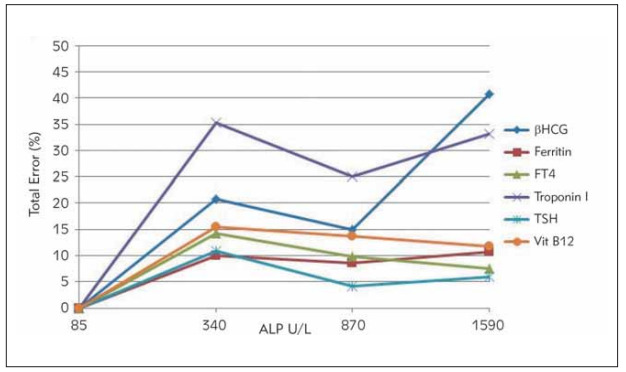
Total error distributions of βhCG, Ferritin, FT4, Troponin I, TSH and Vit B12 tests at different ALP concentrations.Groups containing different ALP concentrations are shown on the x-axis and total error values on the y-axis.

**Figure 2:**
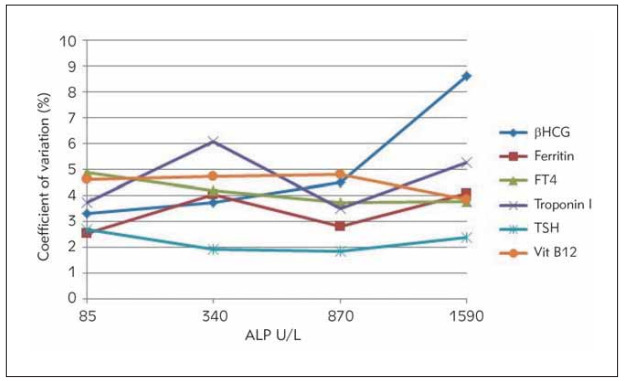
Coefficient of variations of each test at different ALP concentrations. Groups containing different ALP concentrationsare shown on the x-axis, and the calculated coefficient of variations on the y-axis.

CV, Bias, and TE values of each analyte are reported as separate groups in Table 1. ALP interference was assessed by calculating CV, bias, and TE for each analyte and comparing it to allowable total error [9]. Results for each sample pool are summarized in Table 1. Figure 1 shows the TE values of βhCG, Ferritin, FT4, troponin I, TSH and Vit B12 between the groups, while Figure 2 shows the calculated CV's.

**Table 1:** ALP, alkaline phosphatase; aTE, allowable total error; CV, coefficient of variations; FT4, free thyroxine; TE, total error; TSH, thyroid-stimulating hormone; Vit B12, cobalamin; The values marked as bold indicate TE values that exceed the upper limit that recommended by Rili-BAEK [9].

	ALP (85 U/L)	ALP (340 U/L)			ALP (870 U/L)			ALP (1590 U/L)			
Test Name	Mean (CV)	Mean<br>(CV)	Bias %	TE	Mean<br>(CV)	Bias %	TE	Mean<br>(CV)	Bias %	TE	aTE
βHCG, IU/L	0.68 (3.29)	0.77<br>(3.74)	13.23	20.71	0.72<br>(4.50)	5.88	14.88	0.84<br>(8.63)	23.5	40.76	17.00
Ferritin, pmol/L	107.57 (2.55)	105.46<br>(4.02)	-1.96	10.00	104.42<br>(2.82)	-2.92	8.56	104.62<br>(4.06)	-2.73	18.85	13.50
FT4, pmol/L	10.94 (4.88)	11.58<br>(4.2)	5.88	14.28	11.19<br>(3.73)	2.35	9.81	10.94<br>(3.76)	0.05	7.57	13.00
Troponin I, ng/L	19.78 (3.74)	15.19<br>(6.07)	-23.2	35.34	16.20<br>(3.5)	-18.02	25.02	15.29<br>(5.29)	-22.6	33.18	20.00
TSH, mIU/L	2.43 (2.70)	2.46<br>(1.92)	6.99	10.83	2.44<br>(1.85)	0.40	4.10	2.40<br>(2.39)	-1.23	6.01	13.50
vitB12, pmol/L	160.10 (4.60)	169.69<br>(4.72)	5.99	15.43	166.74<br>(4.80)	4.14	13.74	166.74<br>(3.84)	4.14	11.82	25.00

After adding ALP, in βhCG, calculated TE values at ALP concentrations of 340 U/L and 1590 U/L were found to be above the acceptable error limits. In the FT4 test, the calculated TE value at ALP concentrations of 340 U/L was found to be above the acceptable error limits. Calculated TE values were found to be above the acceptable error limits in all groups for the troponin I test. We observed that the calculated CV values for the βhCG test increased with increasing ALP concentrations. In the troponin I test, calculated CV's at ALP concentrations of 340 U/L and 1590 U/L were found to be higher than the group without ALP added.

Table 2 shows the p values obtained by comparing the groups with different ALP concentrations with the group containing baseline ALP. We observed that there were statistically significant differences in all groups for βhCG and Vit B12, in the concentration of ALP 340 U/L for FT4, and in concentrations of 340 and 870 U/L for troponin I when compared to the baseline ALP level (P<0.0125). There were no statistically significant differences in ferritin and TSH among the groups.

**Table 2:** P-values of statistical association analysis by Friedman test with Bonferroni correction for comparison of ALP effects oneach test. P<0.0125 is considered statistically significant.

ALP (85 U/L)		ALP (340 U/L)	ALP (870 U/L)	ALP (1570 U/L)
	βHCG, IU/L	P<0.0125	P<0.0125	P<0.0125
	Ferritin, pmol/L	p>0.0125	p>0.0125	p>0.0125
	FT4, pmol/L	P<0.0125	p>0.0125	p>0.0125
	Troponin I, ng/L	P<0.0125	P<0.0125	p>0.0125
	TSH, mIU/L	p>0.0125	p>0.0125	p>0.0125
	vitB12, pmol/L	P<0.0125	P<0.0125	P<0.0125

## Discussion

In our study, ALP interference was observed in immunoenzymatic assays for bhCG, FT4, troponin I and Vit B12 tests. We observed that TE and CV values increased after ALP addition, especially in troponin I and βhCG tests. Likewise, Sofronescu et al. [10] observed that their patient who used ALP enzyme externally for treatment had low Total testosterone levels than usual. They decided to measure total testosterone by liquid chromatography-mass spectrometry for comparison and found the result in the normal range. They observed negative interference as we observed in the troponin I test. They concluded that ALP could potentially interact and cause interference after binding the antibody [10]. There are some similarities between their study and ours. They used the same auto-analyzer and manufacturer's kit as us. But they calculated neither TE nor CV. At the end of their study, they mentioned inadequate washing of the unbound analyte could also lead to false results [10]. Similarly, Herman et al. [5] reported that βhCG and troponin I measurements were found to be incorrectly high as a result of improper washing steps of samples containing elevated ALP. Herman et al. [5] reported that this effect was seen especially above ALP> 1000 U/L concentrations.

We observed that ALP addition could cause interference on βhCG, FT4, troponin I and Vit B12 tests. While Herman et al. [5] found an erroneous elevation in both of the tests after the addition of ALP, we found an erroneous elevation in the βhCG test and an erroneously low reading in the troponin I test. We used Access II auto-analyzer for these tests, while Herman et al. [5] used DXI-800 auto-analyzer. Although Access II and DXi-800 use the same kits and calibrators and are manufactured by the same manufacturer, they are systems whose operation performances are different from each other. These systems perform the washing process in three cycles by using special washing solutions and eliminate any unbound molecules from the medium by creating a magnetic field using a magnet. Three critical compo-nents of the system are pipetting, washing, and checking the luminometer. If system updates and weekly maintenance are skipped, and the washing performance of the autoanalyzer is not working efficiently, high ALP values may cause interference.

In a way similar to our study, Dasguptaet et al. [11] evaluated ALP interference on troponin I assayed by microparticle immunoassay (MEIA) and fluorometric immunoassay (FIA). They did not observe ALP interference in the MEIA method, while they observed that interference increased with the elevation of ALP enzyme concentrations in the FIA method [11]. Similarly, Butch et al. [12] demonstrated the interference of endogenous ALP on troponin I measurement by the FIA method. They evaluated and reported that the reason for this interference may be related to the washing performance of the system they use; they contacted the device manufacturers and reported that the interference was reduced by improving the washing steps [12].

Similar to our study, Marinheiro et al. [13] compared two troponin I methods using ALP as conjugate (Beckman Coulter Access AccuTnI+3®) and acridinium as conjugate (Abbott Architect STAT high sensitive TnI®). They reported that troponin I was falsely higher in the method which uses ALP. Interestingly, like in our case, the ALP level was normal in their case report. They concluded that endogenous ALP may interfere with the assay by interacting with microparticles even if it is in the normal reference range [13].

While preparing the study plan, we had to determine the final ALP concentration that we would reach. The linearity upper limit of the system that we used (Beckman AU5800) was set at 1500 U/L for the ALP test. Nargis et al. [14] analyzed patients with persistent ALP elevations in their study. They have reported that in the population they studied, ALP values were above 3000 U/L in only 3% of the patient group [14]. So, we decided to stay within the values that we might encounter clinically in the daily workflow. Although we could elevate the ALP levels to much higher levels by adding ALP externally, we preferred to keep our upper limit within the linearity limits of the assay as we did not want to exceed the general patient population.

The interference effect of the presence of heterophilic antibodies on the test for immunoenzymatic methods is reported as a generally erroneous test result [15]. One of the limitations of our study is that we did not examine the samples used in the serum pool for the presence of heterophilic antibodies. If some of the selected samples had contained heterophilic antibody would have possibly affected our entire pool. To avoid this, we could have used heterophilic antibody inhibitors. However, since we did not want to create a different interference source by using a heterophilic antibody inhibiting tube, we ignored the presence of heterophilic antibodies.

Other studies investigating ALP interference areoften presented as case reports and are not statistically strong. They tried to understand and show the interference through a patient. Our study design was structured very well, and we compared our results to the reported guideline.

Based on the findings obtained from our study, we determined that elevated ALP caused interference on βhCG, FT4, troponin I and Vit B12 tests, but it did not cause a significant interference on Ferritin and TSH tests. Especially in terms of misdiagnosing myocardial infarction, it should be considered that troponin I could be affected by high ALP levels. It would be beneficial to repeat the βhCG and troponin I tests with DXI-800. There is a need for repeating the study with samples free of any heterophilic antibodies and with samples containing higher rates of ALP.

## Dodatak

### Conflict of interest statement

All the authors declare that they have no conflictof interest in this work.

### List of abbreviations

ALP, Alkaline phosphatase;<br>βhCG, betahuman chorionic gonadotropin;<br>FT4, Free thyroxine;<br>TSH, thyroid-stimulating hormone;<br>Vit B12, Cobalamin;<br>TE, Total Error;<br>CV, coefficient of variations;<br>MEIA, microparticle immunoassay;<br>FIA, fluorometric immunoassay
